# Simulation Competition Enhances Emergency Manual Uses During Actual Critical Events

**DOI:** 10.7759/cureus.3188

**Published:** 2018-08-23

**Authors:** Jeffrey Huang, Anamaria Parus, Jiayan Wu, Chunyuan Zhang

**Affiliations:** 1 Anesthesiology, University of Central Florida College of Medicine , Orlando, USA; 2 Anesthesiology, University of Central Florida, Orlando, USA; 3 Anesthesiology, Zhongshan Traditional Chinese Medicine Hospital, Zhongshan, CHN; 4 Anesthesiology, Zhongshan Boai Hospital, Guangdong, CHN

**Keywords:** emergency manuals, competition, simulation, survey

## Abstract

Background

Emergency manuals (EMs) are guides that provide a sequence of procedures and are used in response to critical events in the operating room. Literature has described the clinical advantage of such cognitive aids but implementation remains a problem because habits can be difficult to change. Studies have shown that successful use of EMs can be achieved by simulation training. This form of active learning engages the participants and provides the advantage of hands-on experience. Therefore, a simulation competition, namely Simulation Wars, was established in China to promote simulation training and increase training participation. This study aims to assess whether participation in such a simulation competition increases the participants’ implementation of EMs in the operating room in real situations of crisis.

Methods

Anesthesia providers who participated in the 2017 Zhongshan Emergency Manuals simulation training competition and multi-institutional survey studies were eligible to participate in this study. A year after the inaugural competition, surveys were distributed to the participating providers to assess their use of EMs in the operating room post competition.

Results

One hundred six anesthesia providers across two different hospitals qualified for the study. The response rates among anesthesia providers were similar for both surveys, with 45/51 (88.2%) pre-competition and 48/55 (87.2%) post-competition. Analysis shows that EM usage during critical events increased significantly following a simulation training competition (p<0.05).

Conclusion

The study indicates that EM use in the operating room significantly increased after participating in the simulation training competition.

## Introduction

The effective rescue of a patient while in the operating room relies on timely recognition and effective management of the complication [1]. A recent study demonstrated that providers respond to these crisis situations more efficiently by utilizing operating room (OR) emergency manuals (EMs) [2]. These manuals provide a sequence of procedures that are consistent with established medical guidelines in response to a list of critical events [2]. While these clinical emergencies are rare, timely recognition and knowing exactly how to respond can mean the difference between life and death for these patients.

There are several publications that illustrate the clinical advantage of using cognitive aids like EMs. Dr. Ranganathan and colleagues described a case where a four-month-old was saved from malignant hyperthermia with the aid of an EM [3]. Dr. Huang described the resuscitation of a 44-year-old male who developed bronchospasm during a laparoscopic left hepatic lobectomy and cholecystectomy [4]. Utilization of an EM led to rapid management of the patient [4].

Dr. Goldhaber-Fiebert proposed a four-step method to successfully implement EMs as follows: creation of content and design, familiarization with the content by training, usage of EMs, and lastly integration into the culture of the OR [5]. However, despite a growing body of evidence in favor of EM use, changing human behavior can be challenging, especially when dealing with entire groups or systems [6]. Clinicians who are not accustomed to using checklists and guides are less likely to use them in situations of crisis, unless adequate training is provided [7].

One way to increase routine use of EMs is through widespread participation in simulation training events. Studies showed simulations have resulted in successful use of EMs during clinical critical events as reported by anesthesiology residents, with many of them agreeing that EM self-review and immersive simulations positively influenced their subsequent EM use [2, 8].

In order to facilitate implementation of anesthesia emergency manuals in China, Stanford Operating Room Emergency Manuals, Harvard Ariadne Lab Operating Room Crisis Checklists, and Society of Pediatric and Anesthesia Pedicrisis Critical Events Cards were translated into Chinese [8]. These manuals were published (December 25, 2015) in the largest anesthesia network in China, namely New Youth Anesthesia Forum, and 125,000 copies were downloaded within the first six months of publication [8]. Additionally, official Chinese anesthesia organizations endorsed the implementation effort [8]. A year after publication, multi-institutional surveys were distributed to Chinese anesthesia providers and showed that significantly higher levels of EM use were associated with anesthesiologist participation in simulation training, as well as EM group studies and self-review [8]. Additionally, this study concluded that training in a high fidelity simulation center and training in the OR led to similar results, concluding that having access to high fidelity simulation centers may not prove necessary [8]. This means that even facilities with limited resources can successfully conduct simulation training in the OR, which does not require sophisticated or costly equipment [9].

Simulation Wars is a competition created in China whose aim is to promote simulation training and increase training participation [10]. In 2017, the Zhongshan City Society of Anesthesiology invited hospitals to participate in the simulation competition [10]. Participating hospitals submitted a self-written and a self-directed video demonstrating the application of the Stanford Operating Room Emergency Manuals in an anesthesia-related emergency [10]. Each hospital was instructed to focus their simulations on crisis resource management skills by following the EMs [10]. They subsequently performed in-person crisis management performances during the competition’s final round [10].

Following the inaugural event, each participant was asked to complete a survey [10]. Results show that 93% of participants agreed that this competition could increase participation in simulation training [10].

The simulation competition can increase participants’ understanding of how and why to use EMs. To identify the long-term effect of the simulation competition, we distributed a survey to the participating anesthesia providers one year after the competition. The survey aims to assess whether participation in the competition increased the use of EMs in the OR.

## Materials and methods

Hospitals that participated in the 2017 Zhongshan Emergency Manuals simulation training competition and multi-institutional survey studies were included in the study. Therefore, the data from the multi-institutional survey studies were used as a baseline for comparison. Zhongshan Traditional Chinese Medicine Hospital and Zhongshan Boai Hospital met the criteria and were invited to participate in this study. Consent for this study was obtained from the Zhongshan Traditional Chinese Medicine Hospital authority. The survey questionnaires were sent to all anesthesiologists in these hospitals.

For this study, ‘self-review’ was defined as studying the EM oneself. Information on the total number of anesthesiologists in each participating department was collected. In order to protect the confidentiality of the physicians, we did not collect identifying information from the respondents. No patient-identifiers were collected. The survey questionnaire was adapted and modified from the survey used in the Stanford study [2] by the author. It was then distributed to all respondents through WeChat using the Wenjuanxing software.

To maximize total responses, the survey did not contain any open-ended questions. Survey questions included the 5-point Likert scale (strongly disagree to strongly agree), Yes or No boxes, and multiple-choice answers. No monetary compensation was provided for any kind of participation in this survey. Emergency manual use reports for clinical critical events by event type were listed as “check all that apply.”

Comparisons between the groups that used EMs during critical events between 2017 and 2018 was done using the Chi square test. All tests were two-tailed with a type I error rate of 0.05. For quantitative variables, mean and standard deviation (SD) were used.

## Results

Two hospitals (Zhongshan Traditional Chinese Medicine Hospital and Boai Hospital) qualified for the study. A year after the simulation competition, a survey was sent to each anesthesiologist in these two facilities. The data were combined together from the two hospitals. The response rates among anesthesia providers were similar for both surveys, with 45/51 (88.2%) pre-competition and 48/55 (87.2%) post-competition.

The participant demographic data is shown in Table 1. There were no statistically significant differences in the sex of respondents for the pre- and post-surveys, and no statistically significant differences in the respondents’ years of work experience between the survey years.

**Table 1:** Participant demographics

	2017	2018
Male	63%	65%
Female	37%	35%
Chief Physicians	15	15
Attending Physicians	17	16
Resident Physicians	13	17

The frequency of self-review and reported EM usage of each hospital are shown in Table 2. Over 85% of all respondents reported using EM in at least one OR critical event in these two hospitals a year after the simulation competition. Analysis shows that EM usage during critical events increased significantly following a simulation training competition (p<0.05). The frequency of self-review did not change, suggesting that the simulation competition directly contributed to the participants’ willingness or ability to use EMs.

**Table 2:** Frequency of self-review participation and EM use during critical events EM - emergency manual

Hospital	Participated in EM group study or self-review at least once within the last year		Used EMs during critical events at least once within the last year	
2017	yes	No	Yes	no
A	19	0	13	6
B	24	2	17	9
2018				
A	16	3	15	4
B	27	2	26	3

**Figure 1:**
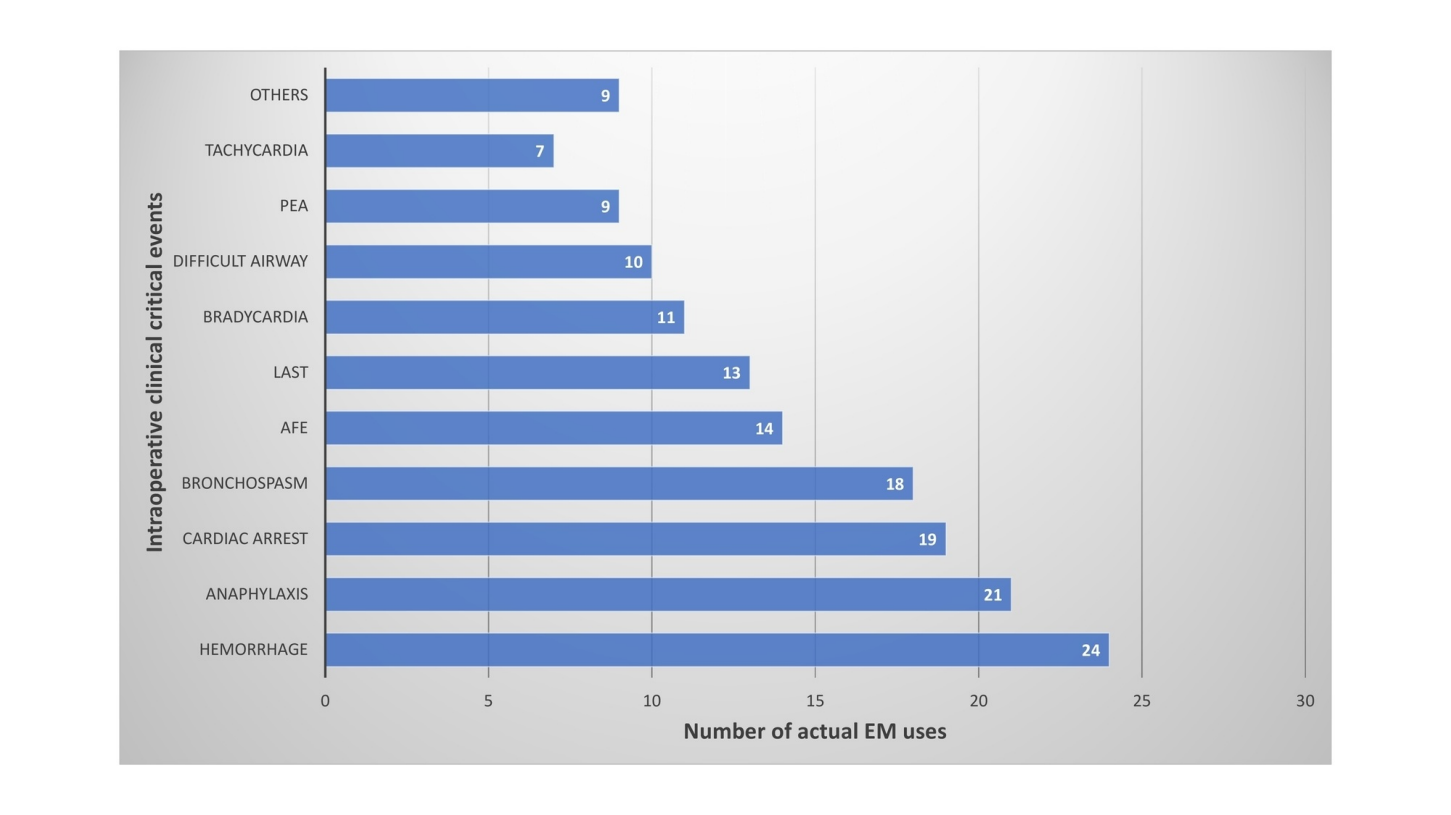
Emergency manual use reports for clinical critical events by event type. Others included the following: oxygen failure, pneumothorax, power failure, transfusion reaction, venous air embolism, hypotension, hypoxemia, delayed emergence, fire, malignant hyperthermia, total spinal anesthesia LAST - local anesthetic toxicity, AFE: amniotic fluid embolism, PEA: pulseless electrical activity

Emergency manual use for clinical critical events by event type is shown in Figure 1. All event types were classified in the Stanford Emergency Manual for Perioperative Critical Events, by Stanford Anesthesia Cognitive Aid Group (SACAG) [11]. The manual contains 25 critical events as well as Crisis Resource Management key points [11]. The manual is available at http://emergencymanual.stanford.edu. The cardiac arrest category includes multiple ACLS (Advanced Cardiac Life Support) event types [11]. The following event types were not reported as used in this survey: oxygen failure, pneumothorax, power failure, transfusion reaction, and venous air embolism.

## Discussion

The study showed that the emergency manual Simulation Wars can promote simulation training and subsequently increase the use of EMs in crisis situations in the OR. While the frequency of pre-competition and post-competition self-review was not statistically different, our study indicates that EM use in the OR significantly increased after participating in the simulation training competition.

Semler and colleagues performed a study that compared teamwork in a simulation based environment versus traditional didactic methods and showed that simulation based training was superior in attaining teamwork skills [12]. Another study showed that surgeons who performed simulation training on a virtual reality device were more efficient, had better skills and committed less errors while performing endoscopic sinus surgery than those that did not have the simulation training [13]. These concepts of increased efficacy with the aid of simulation training can be similarly applied to this study, in which the simulation competition leads to familiarization with the EM content by means of hands-on experience. The more familiar providers are with the EM, the more comfortable they are in using it in situations of crisis in the OR. Although change of habits can be hard to implement, frequent practice and increased use of EMs during crises can lead to a culture where their use becomes second nature and EMs are integrated in the OR culture. Increasing one’s EM knowledge is vital to its integration, as physicians perform better in managing simulated cardiac arrests after they are trained to use ACLS cognitive aids [10]. Familiarization of the team members with the EM location, content, and application is one of the four key steps towards proper integration into operating rooms [5]. The gamification of learning in Simulation Wars makes the learning experience more engaging. Each team designs a unique simulation scenario and demonstrates the proper application of an emergency manual. Participants have to write the scenario, organize the team, assign a leader, practice leading the team, give each other feedback, and follow the emergency manuals to relieve the crisis. They practice multiple times before submitting the video and performing in the final round. During the final round, participants also observe the performance of other teams, supplementing their own hands-on experience by learning from their colleagues.

While participation in simulation competition has its advantages in providing hands-on experience, Semler and colleagues demonstrate that teamwork training with expert demonstration produces similar results as participating in simulations, while both methods are superior to didactic instruction [12]. Expert demonstration is another learning tool that could be explored in the future to aid implementation of EM, as it has proven to be more cost effective than simulation [12].

Although there are only a handful of providers in the competition, these providers can become the clinical champions in their department, leading and training others to use EMs. Clinical champions are individuals who advocate tenaciously for a cause in the workplace, who serve as team leaders and motivate staff [14]. They are also in control of educating and training staff about an initiative, recruiting team members for implementation, utilizing data for persuasion, as well as troubleshooting problems that emerge during the implementation process [14].

Studies on the success of clinical champions are few and have mixed results. One study performed in Canada showed that influenza vaccination rates in the champion condition outperformed the training session-only condition (52% vs 41%) [15]. A study performed by McCabe et al. in Australia aimed to improve staff detection of depression in residential care settings [16]. The champion group outperformed the control group (87% vs 43%) and the education-only group (87% vs 52%) in correctly identifying residents with depression [16]. However, the control group and the education-only group outperformed the champion group in correctly identifying no depression in non-depressed patients [16]. This evidence shows that champion implementation might be useful for certain methods of instruction and less useful for others and more research needs to be developed on this subject.

Furthermore, a study conducted by Damschroder et al. found that while it is possible for a single champion to implement a new technology, improvements that require people to change behaviors need more than one champion [17]. Behavioral changes are more complicated because they require interprofessional coalitions to join forces and work together [17]. Similarly, the implementation of EMs in operating rooms requires more than a champion and partly depends on the quality of the organizational network.

Limitations

While China has made some efforts in recent years, it is known that anesthesiology training in China lacks standardization, and therefore the perception of what counts like a critical event may vary [8, 18]. While the critical events should have theoretically fallen within the 23 categories mentioned in the EM, our survey did not provide a clear definition of what constitutes a ‘critical event’ and the criteria might vary from provider to provider.

Our survey asked participants to recall critical events in the past year and due to the nature of this study, accuracy and honesty cannot be verified. Some respondents might have forgotten to recall certain events. Selection bias is another factor, as providers who use cognitive aids might be more likely to report events.

Additionally, since this survey was given out to anesthesia providers at only two hospitals, it is possible that different respondents worked on the same case and some critical events might have been reported more than once. Since this study only included two hospitals, it may be difficult to extrapolate these results to a larger population.

In an effort to keep the questions concise and maximize response, open-ended questions were not included in the survey. Open-ended questions could have provided qualitative data, such as ways to improve simulation competitions to get more participation and reveal factors that led to usage and non-usage of EMs in situations of crisis.

Similarly, while this study demonstrates an increased use of EM in the OR, there is no evidence of improvements in clinical outcomes. The real value of EMs depends on whether it results in improved patient outcomes, which is a topic that needs to be explored in future research.

## Conclusions

The ultimate goal of the simulation competition was to increase EM usage during critical events in the OR. Our study provides evidence that there is an increased use of EMs in real situations of crisis after providers have participated in a simulation competition. These results could be used as evidence to establish a national standard to facilitate implementation of EMs by providing hands-on experience, like simulation competitions. Further research is needed to evaluate the use of EMs and clinical outcomes.

## References

[1] Ghaferi AA, Birkmeyer JD, Dimick JB (2009). Variation in hospital mortality associated with inpatient surgery. N Engl J Med.

[2] Goldhaber-Fiebert SN, Pollock J, Howard SK, Bereknyei Merrell S (2016). Emergency manual uses during actual critical events and changes in safety culture from the perspective of anesthesia residents: a pilot study. Anesth Analg.

[3] Ranganathan P, Phillips JH, Attaallah AF, Vallejo MC (2014). The use of cognitive aid checklist leading to successful treatment of malignant hyperthermia in an infant undergoing cranioplasty. Anesth Analg.

[4] Huang J, Jiang F, Zhang J, Li M (2018). The use of emergency manuals leading to successful treatment of severe bronchospasm: a case report. J Med Pract Manage.

[5] Goldhaber-Fiebert SN, Howard SK (2013). Implementing emergency manuals: can cognitive aids help translate best practices for patient care during acute events?. Anesth Analg.

[6] Hepner DL, Arriaga AF, Cooper JB (2017). Operating room crisis checklists and emergency manuals. Anesthesiology.

[7] Watkins SC (2013). A simulation-based trial of surgical-crisis checklists. N Engl J Med.

[8] Huang J, Wu J, Dai C (2018). Use of emergency manuals during actual critical events in China: a multi-institutional study. Simul Healthc.

[9] Huang J (2016). Implementation of emergency manuals in China. APSF Newsletter.

[10] Zhang C, Zeng W, Rao Z (2017). Assessment of operating room emergency manual simulation training. [Article in Chinese]. Perioperative Safety and Quality Assurance.

[11] Group SACA. Emergency Manual (2018). Group SACA. Emergency manual cognitive aids for perioperative critical events. http://emergencymanual.stanford.edu (accessed June.

[12] Semler MW, Keriwala RD, Clune JK (2015). A randomized trial comparing didactics, demonstration, and simulation for teaching teamwork to medical residents. Ann Am Thorac Soc.

[13] Fried MP, Sadoughi B, Gibber MJ (2010). From virtual reality to the operating room: the endoscopic sinus surgery simulator experiment. Otolaryngol Head Neck Surg.

[14] Miech EJ, Rattray NA, Flanagan ME, Damschroder L, Schmid AA, Damush TM (2018). Inside help: an integrative review of champions in healthcare-related implementation. SAGE Open Med.

[15] Slaunwhite JM, Smith SM, Fleming MT, Strang R, Lockhart C (2009). Increasing vaccination rates among health care workers using unit "champions" as a motivator. Can J Infect Control.

[16] McCabe MP, Karantzas GC, Mrkic D, Mellor D, Davison TE (2013). A randomized control trial to evaluate the beyondblue depression training program: does it lead to better recognition of depression?. Int J Geriatr Psychiatry.

[17] Damschroder LJ, Banaszak-Holl J, Kowalski CP, Forman J, Saint S, Krein SL (2009). The role of the champion in infection prevention: results from a multisite qualitative study. Qual Saf Health Care.

[18] Lio J, Dong H, Ye Y, Cooper B, Reddy S, Sherer R (2016). Standardized residency programs in China: perspectives on training quality. Int J Med Educ.

